# β-lactam resistance in bacteria associated with subclinical mastitis in goats in Thika Subcounty, Kenya

**DOI:** 10.14202/vetworld.2020.1448-1456

**Published:** 2020-07-25

**Authors:** Irene Mkavi Okoko, Naomi Maina, Daniel Kiboi, John Kagira

**Affiliations:** 1 Department of Molecular Biology and Biotechnology, Pan-African University, Institute of Basic Sciences, Technology and Innovation, P.O. Box 62000-00200, Nairobi, Kenya; 2 Department of Biochemistry, Jomo Kenyatta University of Agriculture and Technology, P.O. Box 62000-00200, Nairobi, Kenya; 3 Department of Animal Sciences, Jomo Kenyatta University of Agriculture and Technology, P.O. Box 62000-00200, Nairobi, Kenya

**Keywords:** bacteria, dairy goats, Kenya, subclinical mastitis, β-lactam resistance

## Abstract

**Aim:**

This study determined the resistance pattern to β-lactam antibiotics of bacteria isolated from goats with subclinical mastitis in Thika subcounty, Kenya. We also administered a questionnaire to assess the risk factors associated with the occurrence of resistance to commonly used antibiotics.

**Materials and Methods:**

We collected milk samples from 110 lactating dairy goats in Thika subcounty to screen for subclinical mastitis using the California mastitis test. Bacterial isolation and identification were performed according to colony morphology, the hemolytic pattern on sheep blood agar, lactose fermentation on MacConkey plates, Gram staining, and standard biochemical tests. The antibiotic susceptibility of the isolates was determined by the agar disk diffusion method using penicillin G, cephalexin, cefoxitin, and cefotaxime antibiotic disks. The double-disk synergy test using amoxicillin-clavulanic acid was employed as a confirmatory test for extended-spectrum β-lactamase (ESBL) production. Fisher’s exact test was used to determine the risk factors associated with the occurrence of antibiotic resistance (p≤0.05 was considered significant).

**Results:**

Of the 110 dairy goats sampled, 72.7% (80) were positive for subclinical mastitis. Isolation and identification of the bacteria from the positive samples yielded 149 bacteria isolates, including *Staphylococcus aureus*, *Klebsiella pneumoniae*, *Acinetobacter* spp., *Yersinia* spp., coagulase-negative staphylococci, and *Escherichia coli*. A high percentage (76.5%, 114/149) of the bacterial isolates was resistant to at least one of the tested antibiotics. At least 56/106 isolates (52.8%) showing cross-resistance to the β-lactam antibiotics were resistant to all four of the tested antibiotics, while only one isolate was resistant to three antibiotics (penicillin G, cephalexin, and cefoxitin). The double-disk synergy test confirmed that none of the isolates possessed ESBLs. Pre- and post-milking practices (p=0.0336) were found to be significantly associated with the occurrence of antibiotic resistance.

**Conclusion:**

A large proportion of the goats in our study cohort were infected with β-lactam-resistant bacteria associated with subclinical mastitis. Because the identified bacteria are of zoonotic importance, further studies should be undertaken to determine the transmission dynamics between humans and livestock and to identify novel intervention strategies.

## Introduction

The dairy goat agricultural industry is rapidly expanding. In 2017, the global dairy goat population was estimated at 218 million, producing approximately 18.7 million tons of milk [1]. Sub-Saharan African countries account for a goat population of 372,716,040, with 16% farmed as dairy goats [2]. The dairy goat population in Kenya was estimated as >251,100 in 2011, with the milk used as an alternative to cow’s milk. At present, approximately 4.7% of the total milk consumed in Kenya is obtained from goats [3]. The emerging interest in goats and their derivative products, including milk, is due to the associated health and nutritional benefits, low production cost, ease of management, and goat dairy farming being a source of livelihood, among other factors [1,3,4]. Despite the vast interest in dairy goat production, the enterprise faces a myriad of abiotic and biotic challenges. Of major concern is mastitis, a disease resulting from the infection of mammary glands by viruses, fungi, or bacteria, with the latter being the major etiological agents [5]. Following mammary gland infection, white blood cells and epithelial (somatic) cells are produced by the goat as a defense mechanism [6]. A relatively small number of epithelial cells are present in milk from healthy milk-secreting tissue (1,000,000 cells/ml), and somatic cell counts (SCC) >1,500,000 cells/ml indicate intramammary infection [6]. Mastitis is associated with symptoms such as udder inflammation and tissue damage, leading to reduced milk quality and quantity [7]. Mastitis manifests either clinically (symptomatic) or subclinically (asymptomatic), the latter being more prevalent in goats [8]. Coupled with the treatment cost, disease management has led to significant economic losses [9]. The mastitis management strategies in Kenya entail the use of antibiotics, including tetracyclines, β-lactams, trimethoprim/sulfamethoxazole, nitrofurans, aminoglycosides, and quinolones [10], the majority of which belong to the β-lactam family, such as penicillin and cephalosporins [11]. β-lactam antibiotics share a common structural feature, the β-lactam ring, that inhibits bacterial cell wall synthesis [12].

Reports of bacterial resistance to β-lactam antibiotics in cases of mastitis have recently emerged [13-15] and have been attributed to the ability of the bacteria causing mastitis to produce β-lactamase enzymes and modified penicillin-binding proteins that hydrolyze or inhibit the binding of β-lactam antibiotics, respectively [12,16]. Antibiotic resistance in pathogens that cause subclinical mastitis in animals poses a risk of the transfer of antibiotic resistance to humans, especially where raw milk and raw milk products are consumed, such as in Kenya [17,18]. Antibiotic-resistant bacteria cause severe infections with limited treatment options and fatalities, leading to a high cost of treatment, loss of productivity, and adverse economic consequences in the affected country [19,20]. Although a Kenyan study reported resistance to penicillin G in 100% of the bacterial isolates causing subclinical mastitis in goats [21], the resistance of mastitis-causing pathogens to other β-lactam antibiotics remains undocumented in Kenya.

This study determined the prevalence of bacterial resistance to selected β-lactam antibiotics in cases of subclinical mastitis using the agar disk diffusion method. We also report the risk factor associated with the occurrence of resistance to these antibiotics. The study highlighted the consumption of raw milk as a potential vehicle for the transfer of bacterial resistance to humans.

## Materials and Methods

### Ethical approval

Since no invasive procedure was performed on the animals, approval from the Institutional Animal Ethics Committee to conduct the study was not required.

### Study area and period

The study was conducted in Thika subcounty of Kiambu County, located in Central Kenya, approximately 40 km from Nairobi (1°1’S 37°5’E). Rainfall is bimodal, ranging from 500 to 1300 mm, and the average temperature is 18.7°C. The study was conducted for a period of 10 months (January 2019 to October 2019). Although no formal census has been undertaken, farmers have taken up dairy goat farming in this region [21]. Farmers practice zero-grazing, open-grazing, and tethering-grazing systems [Personal communication, Veterinary officer, Thika subcounty].

### Study design, sample size calculation, and questionnaire administration

A cross-sectional field- and laboratory-based study design was used. The sample size of 110 lactating dairy goats was calculated using an adjusted formula for small populations [22], whereby the sample size for an infinite population with an estimated prevalence can be calculated for a 95% confidence interval as n=(1.96^2^[P_exp_][(1−P_exp_])/d^2^, where *n* is the required sample size, P_exp_ is the expected prevalence, and *d* is the desired absolute precision. We used a prevalence of 50.9% subclinical mastitis, which was determined in a study of goats in Thika East subcounty [21], and a desired absolute precision of 5%, resulting in a required sample size of 384 goats. In relatively small populations, such as that of dairy goats in Thika subcounty, it is possible to select a smaller sample size than one from a theoretically infinite population to achieve the same degree of precision using the adjusted formula N_adj_=N×n/N+n, where n is the sample size as calculated above and N is the size of the study population [22]. Given that the size of the study population of lactating dairy goats was approximately 150 [21], a sample size of 108 does was calculated. We sampled 110 does.

We targeted small-scale farmers with a herd size of ≤15 goats who consented to participate. Sampling was performed using the snowball technique and sampling to redundancy due to the absence of a formal list of dairy farmers in the area [23]. The local agriculture extension officer assisted in identifying the first farmers, who later recruited other farmers until all the farmers had been included. The final cohort comprised 110 lactating goats with no clinical signs of mastitis, sampled from 41 farms. We administered a questionnaire to the farmers to collect data pertaining to age, parity, mastitis history, mastitis management, cleaning schedules of the housing structure, pre- and post-milking practices, and other variables.

### Milk sampling and California mastitis test (CMT)

The milk samples were aseptically collected, as previously described [21]. CMTs (ImmuCell, ME, USA) were performed to screen for subclinical mastitis, according to the manufacturer’s instructions with minor modifications. Briefly, 5 ml of milk from different teats were milked into the CMT paddle, and an equal volume of the CMT reagent was added. A positive CMT test was scored as 2 or 3, indicating an SCC of 2,560,000 and ≥10,000,000, respectively [6]. In addition, 5 ml of milk from the samples with CMT scores of 2 and 3 were collected into sterile universal bottles, placed in a cooler box containing ice packs, and transported within 12 h of collection to the microbiology laboratory at Jomo Kenyatta University of Agriculture and Technology, Juja, Kenya, for bacteriological culture and isolation.

### Bacterial culture and identification

Sheep blood agar (HiMedia, Mumbai, India) and MacConkey agar (Oxoid, Basingstoke, UK) were freshly prepared as per the manufacturer’s guidelines and inoculated with approximately 100 μl of each milk sample per plate, respectively. In routine microbiological diagnosis, a sample of 10-100 μl of milk is used for culture. However, an inoculum of 100 μl has a higher sensitivity when isolating bacteria causing intramammary infection [24]. Hence, we used 100 μl for inoculation. The milk was evenly spread onto the surface of the agar plate using a sterile glass spreader and then incubated at 37°C for up to 48 h. The morphological characteristics of the resulting bacterial colonies were assessed for shape, color, size, and texture as well as lactose fermentation ability on MacConkey plates and hemolysis patterns on sheep blood agar plates. Hemolysis of the blood cells in the sheep blood agar was evaluated by noting any color change in the media, particularly around and under the colonies. A plate was said to have mixed growth when the colonies on that plate showed distinct morphological characteristics, and each of the distinguishable colonies was then subcultured in nutrient agar to obtain pure colonies. The pure cultures were examined for colony morphology, Gram staining characteristics (to distinguish between Gram-positive and Gram-negative isolates), and biochemical reactions.

A catalase test using 3% hydrogen peroxide was performed on all Gram-positive isolates to identify *Staphylococcus* spp. *Staphylococcus* spp. were also identified based on their growth and fermentative attributes on mannitol salt agar (HiMedia) and the results of an oxidase test (HiMedia). *Staphylococcus aureus* was differentiated from coagulase-negative staphylococci (CNS) using slide and tube coagulase tests that employed rabbit plasma.

The oxidase test was performed on all Gram-negative isolates to identify *Enterobacteriaceae*, and those isolates that were oxidase negative were subsequently subcultured on MacConkey agar to assess lactose fermentation. Then, the triple sugar iron (Oxoid), methyl red/Voges–Proskauer (HiMedia), urease (urea broth base; HiMedia), indole (tryptone broth; HiMedia), citrate utilization (Simmons citrate agar; HiMedia), motility (sulfur indole motility media; HiMedia), catalase, and mannitol salt agar (HiMedia) tests were employed to classify the Gram-negative bacteria. The results of culture, Gram staining, and biochemical tests were used for final bacterial identification [25-27].

### Antibiotic susceptibility testing of the isolates

Antibiotic susceptibility testing was performed using the Kirby–Bauer disk diffusion method [28], and the results were interpreted following the Clinical Laboratory Standard Institute guidelines (https://clsi.org/). Briefly, the bacterial suspension was prepared in sterile distilled water and standardized to 0.5 McFarland’s standard (corresponding to approximately 1.5×10^8^ colony-forming units/ml). Mueller-Hinton agar was prepared following the manufacturer’s instructions, poured into inoculation plates to solidify, and inoculated with the standardized inoculums. *Escherichia coli* ATCC 25922 (American Type Culture Collection, Rockville, MA) was used for quality control. Bacterial resistance was tested using commercially available antibiotic disks of penicillin G (10 μg), cephalexin (30 μg), cefoxitin (30 μg), and cefotaxime (30 μg). These were randomly selected from β-lactam antibiotics commonly used in Kenya to treat bacterial infections in both animals and humans and included different generations and classes of β-lactam antibiotics, with penicillin G as a representative of the penicillin class of β-lactams. Cephalexin, cefoxitin, and cefotaxime were representative of the first-, second- and third-generation cephalosporins, respectively. The disks were placed on the agar surface 24 mm from center to center, and the plates were incubated at 37°C for 18 h. The zones of growth inhibition, including the disk diameters, were measured in millimeters and recorded as resistant, intermediate, or susceptible to the specific antibiotics [29].

### Determining the proportion of extended-spectrum β-lactamase (ESBL) producers

The double-disk synergy test was used for phenotypic confirmation of ESBL producers, as previously described [30]. *E. coli* ATCC 25922 and *E. coli* ATCC 35218 (American Type Culture Collection) were used as negative and positive controls, respectively. Commercially available disks (HiMedia) of cefotaxime (30 μg) and ceftazidime (30 μg) together with amoxicillin-clavulanic acid (30 μg) were placed 25 mm apart from each other on the surface of inoculated Mueller-Hinton agar plates and incubated at 37°C for 24 h. The test was regarded as positive when the decreased susceptibility to cefotaxime and/or ceftazidime was joined with a clear-cut augmentation of the inhibition zone of cefotaxime and/or ceftazidime in front of the amoxicillin-clavulanic acid antibiotic disk, forming a characteristic “champagne-cork” or “keyhole” shape [30]; otherwise, the results were regarded as negative. The bacterial samples were stored in tryptic soy broth (Scharlab SL, Barcelona, Spain) containing 20% glycerol at −70°C for long-term preservation.

### Determining the relationship between the occurrence of resistance against the selected β-lactam antibiotics and doe- and farm-level variables

A well-structured questionnaire was developed based on that of Mahlangu *et al*. [21]. The questionnaire was used to collect data regarding farm-level and doe-level variables, such as mastitis history, previous antibiotic treatment of mastitis, pre- and post-milking practices, and the cleaning schedule of the housing structure as well as the doe name, age, parity, and lactation stage. It was written in English and administered to the farmers in English before sample ­collection. However, based on the farmers’ level of understanding, the agriculture extension officer helped with the translation to Swahili or Kikuyu (the vernacular language used by the local farmers in the study area).

The results of the agar disk diffusion test were used to determine the relationship between the occurrence of resistance against the β-lactam antibiotics and doe- and farm-level variables obtained from the questionnaire.

### Statistical analysis

Data were entered into a Microsoft Office Excel spreadsheet Version 16.0.12827.20336 (Microsoft Corp., Redmond, WA, USA) and then analyzed using StatXact 11 (Cytel Inc., Cambridge, MA, USA) and GraphPad Prism software version 7.0.0 (GraphPad Software, CA, USA). Descriptive statistics were expressed as percentages and frequencies, and data were presented in tables and histograms. The prevalence of subclinical mastitis was determined as the proportion of CMT-positive goats out of the total number of sampled goats. The proportion of the resistant bacterial isolates was determined as the number of all the isolates resistant to at least one of the antibiotics out of the total number of bacterial isolates. The association of the doe- and farm-level variables with the occurrence of resistance was determined by Fisher’s exact test. p≤0.05 was considered statistically significant.

## Results

### Prevalence of subclinical mastitis in goats from Thika subcounty

We sampled 110 lactating dairy goats from 41 farms in Thika subcounty with no more than 15 lactating dairy goats per farm (average, three lactating goats). Subclinical mastitis was present in 80 of the dairy goats sampled (72.7%).

### Bacteria causing subclinical mastitis in goats from Thika subcounty

Bacteria were cultured in 97.5% (78/80) of the CMT-positive milk samples. The volume of 2.5% (2/80) of the samples was insufficient for microbiology culture and analysis after performing the CMT test. We cultured 149 isolates from the raw milk samples. In descending order of occurrence, the isolates included *S. aureus*, *Klebsiella pneumoniae*, *Acinetobacter* spp., *Yersinia* spp., CNS, *E. coli*, *Enterobacter intermedius*, *Klebsiella oxytoca*, *Proteus vulgaris*, *Citrobacter freundii*, *Citrobacter diversus*, *Morganella morganii*, *Proteus mirabilis*, *Serratia fonticola*, *Providencia stuartii*, *Serratia marcescens*, and *Staphylococcus epidermidis* (Table-1).

**Table-1:** The proportion of bacteria isolated from CMT-positive milk samples obtained from goats in Thika subcounty.

Bacterial isolate	No. of isolates n=149	Proportion (%)
*Staphylococcus aureus*	26	17.5
*Klebsiella pneumoniae*	26	17.5
*Acinetobacter* spp.	24	16.1
*Yersinia* spp.	21	14.1
CNS	15	10.1
*Escherichia coli*	6	4.0
*Enterobacter intermedius*	5	3.4
*Klebsiella oxytoca*	4	2.7
*Proteus vulgaris*	4	2.7
*Citrobacter freundii*	3	2.0
*Citrobacter diversus*	3	2.0
*Morganella morganii*	3	2.0
*Proteus mirabilis*	2	1.3
*Serratia fonticola*	2	1.3
*Providencia stuartii*	2	1.3
*Serratia marcescens*	2	1.3
*Staphylococcus epidermidis*	1	0.7
Total	149	

CNS=Coagulase-negative staphylococcus, CMT=California mastitis test

### Resistance profiles of bacteria isolated from CMT-positive milk samples from goats in Thika subcounty to the selected β-lactam antibiotics

**Figure-1:**
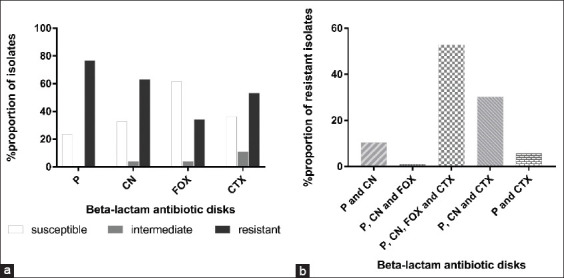
Resistance pattern of the bacterial isolates to beta-lactam antibiotics. (a) Overall susceptibility profiles of the bacterial isolates from CMT-positive milk samples to penicillin G, cephalexin, cefoxitin, and cefotaxime. (b) Cross-resistance of the bacterial isolates from CMT-positive milk samples to the selected beta-lactam antibiotics. P=Penicillin G, CN=Cephalexin, FOX=Cefoxitin, CTX=Cefotaxime.

The disk diffusion tests showed that 23.5% (35/149) of the bacterial isolates were susceptible to all the antibiotics tested, and 76.5% (114/149) showed intermediate or complete resistance to at least one β-lactam. The susceptibility of the isolates to penicillin G, cephalexin, cefoxitin, and cefotaxime was 23.5%, 32.9%, 61.7%, and 36.2%, respectively. Intermediate resistance of the bacterial isolates to penicillin G, cephalexin, cefoxitin, and cefotaxime was 0%, 4.0%, 4.0%, and 10.7%, respectively. Complete resistance to penicillin G, cephalexin, cefoxitin, and cefotaxime was 76.5%, 63.1, 34.2%, and 53.0%, respectively (Figure-1a).

Cross-resistance to the β-lactam antibiotics was observed in 93.0% (106/114) of the resistant bacterial isolates, with 10.4% (11/106) resistant to penicillin G and cephalexin; 0.9% (1/106) resistant to ­penicillin G, cephalexin, and cefoxitin; and the majority (52.8%, 56/106) resistant to penicillin G, cephalexin, cefoxitin, and cefotaxime. Resistance to penicillin G, cephalexin, and cefotaxime was observed in 30.2% (32/106) of the isolates, while 5.7% (6/106) showed resistance to penicillin G and cefotaxime (Figure-1b). In general, a larger proportion of the bacteria resistant to penicillin G showed cross-resistance to other β-lactam antibiotics (Table-2).

**Table-2:** Resistance to penicillin G, cephalexin, cefoxitin, and cefotaxime of the predominant bacterial isolates in CMT-positive milk samples from goats in Thika subcounty.

Antibiotic	% proportion of resistant bacterial isolates					

	*Staphylococcus aureus* (n=26)	*Klebsiella pneumoniae* (n=26)	*Acinetobacter* spp. (n=24)	*Yersinia* spp. (n=21)	CNS (n=15)	*Escherichia coli* (n=6)
P	26.9	0	0	4.8	0	0
P and CN	0	3.8	8.3	4.8	6.7	33.3
P, CN, and FOX	3.8	0	0	0	0	0
P, CN, FOX, and CTX	38.5	34.6	54.2	33.3	40	16.7
P, CN, and CTX	3.8	42.3	4.2	19	20	33.3
P and CTX	0	3.8	8.3	0	20	0

CNS=Coagulase-negative staphylococcus, P=Penicillin G, CN=Cephalexin, FOX=Cefoxitin, CTX=Cefotaxime

### Non-ESBL-producing bacterial isolates from CMT-positive milk samples from goats in Thika subcounty

The double-disk synergy test did not show any characteristic “champagne-cork” or “keyhole” inhibition zones. Thus, this phenotypic method confirmed that none of the bacterial isolates were ESBL producers.

### Relationship between the occurrence of resistance to the selected β-lactam antibiotics and doe- and farm-level variables

In terms of a history of mastitis, higher resistance to the selected β-lactam antibiotics occurred in the does that had previous cases of mastitis compared with no mastitis history, but this relationship was not statistically significant (p=0.4242). A higher prevalence of β-lactam resistance occurred in does that were administered antibiotics when they had mastitis compared with those where no treatment was received, but there was no significant association (p=0.5781) between antibiotic treatment and the occurrence of resistance (Table-3).

**Table-3:** Effects of history of mastitis and antibiotic treatment on occurrence of resistance to the selected beta-lactam antibiotics in dairy goats with subclinical mastitis in Thika subcounty.

Risk factor	Frequency of positive resistant	% proportion of positive resistant	p-value
History of mastitis			
Yes	21/23	91.3	0.4242
No	46/55	83.6	
Antibiotic treatment			
Treatment administered	17/19	89.5	0.5781
No treatment administered	3/3	100	

The occurrence of resistance to β-lactams was significantly affected by the pre- and post-milking practices adopted by the farmers in the region (p=0.0336). The highest prevalence (94.1%) of resistance was observed in bacteria isolated from does where the farmers only used water and a reusable towel for cleaning before and after milking. There was a lower prevalence (77.8%) of resistance in does where the farmers used water, disinfectant, and a reusable towel, while no resistance (0.0%) was observed where the farmers used water and a disposable towel during milking. The highest resistance (100.0%) occurred in does whose housing structures were cleaned every 2 days, after 2 weeks, 3 times a week, or irregularly compared with those that were cleaned once a week (81.8%), twice a week (81.8%), and daily (87.5%). No resistance was seen in those that were cleaned every 3 days (0.0%). From the data in the questionnaire on goats whose house were cleaned every 3 days, the farmers used water alone and udder and teats were dried with a disposable towel during milking. From the data analysis on milking practices used by the farmers, which was found to be significantly associated with the occurrence of resistance, the goats whose farmers used water alone and udder and teats dried with disposable a towel showed no resistance to beta-lactam antibiotics. Therefore, despite the goats’ housing structure being cleaned every 3 days, the milking practices used on these goats might be a contributing factor in preventing the occurrence of resistance in these goats.

On the other hand, the goats whose houses were cleaned every 2 days had their udder and teats dried using a reusable towel during milking. Therefore, despite the goats’ housing structure being cleaned every 2 days, the milking practices used on these goats might be a factor contributing to the occurrence of resistance in these goats. However, resistance was not significantly affected by the cleaning schedule of the housing structure (p=0.2141) (Table-4).

**Table-4:** Effects of pre- and post-milking practices and cleaning schedule on occurrence of resistance to the selected beta-lactam antibiotics in dairy goats with subclinical mastitis in Thika subcounty.

Risk factor	Frequency of positive resistant	% proportion of positive resistant	p-value
Hygiene during milking			
Use of water alone and udder and teat dried with disposable towel	0/2	0	0.0336
Use of water, disinfectant, and udder and teat dried with reusable towel	14/18	77.8	
Use of water alone and udder and teat dried with reusable towel	16/17	94.1	
Use of water alone and udder and teat are not dried	23/26	88.5	
Goat house cleaning schedule			
Once a week	18/22	81.8	0.2141
Daily	21/24	87.5	
After 3 days	0/2	0	
After 2 days	4/4	100	
After 2 weeks	4/4	100	
Twice a week	9/11	81.8	
Thrice a week	2/2	100	
Irregularly	9/9	100	

Resistance was highest among does >4 years old (88.9%) and does with a parity ≤4 (85.7%). Resistance was lowest in does ≤4 years old (86.7%) and does with a parity >4 parity (80.0%). However, there was no significant association between age and parity, respectively, and the occurrence of resistance (p=0.6203 and p=0.333, respectively) (Table-5).

**Table-5:** Effects of age and parity on occurrence of resistance to the selected beta-lactam antibiotics in dairy goats with subclinical mastitis in Thika subcounty.

Risk factor	Frequency of positive resistant	% proportion of positive resistant	p-value
Age			
≤4 years	39/45	86.7	0.6203
>4 years	24/27	88.9	
Parity			
≤4	54/63	85.7	0.333
>4	4/5	80.0	

## Discussion

Mastitis is an economically important disease of dairy goats and cattle. This infection is mainly subclinical in goats, making it impossible to diagnose by physical examination [8]. Losses resulting from subclinical mastitis include decreased milk production and quality and increased veterinary expenses as well as progression to the clinical form [31]. This study determined resistance against penicillin G, cephalexin, cefoxitin, and cefotaxime in bacterial isolates from milk of goats with subclinical mastitis in Thika subcounty. We found a higher prevalence of subclinical mastitis than that reported in Northern region, Bangladesh [32], Tando Jam, Pakistan [33] and Nigeria [34]. However, our result was in agreement with that recorded in the Mount Kenya region, Kenya [35], the state of Minas Gerais, Brazil [36], and Thika East subcounty [21]. Fluctuations in the prevalence of mastitis in different geographical regions are attributed to the complexity of the disease resulting from the interaction of several factors, including management and husbandry strategies, geographical distribution, the herd’s health condition, biological agents causing mastitis, and environmental and nutritional conditions as well as the sample size used in the studies [34].

Our results showed that the highest proportion (28.18%) of the bacterial isolates belonged to the *Staphylococcus* genus, particularly *S. aureus* (17.45%), which was similar to other studies reporting that the highest proportion of the isolates from goats with subclinical mastitis was *S. aureus*, ranging from 23.4% to 61.64% [15,33,37,38]. Staphylococci are listed as the major causative agents in the development of contagious mastitis [5]. This is attributed to the ability of these bacteria to produce highly toxic metabolites that damage the membranes of mammary glands [39]. In addition, *Staphylococcus* is effectively transmitted through several routes [39]. Therefore, in regions where hand milking is mostly practiced, such as Thika subcounty, *Staphylococcus* can be spread during milking operations, which could be associated with its high prevalence. An intramammary infection of *S. aureus* is a public health concern since the bacteria produce thermostable exotoxins that cause toxic shock syndrome and staphylococcal food poisoning. Furthermore, *S. aureus* can also infect humans and animals when unpasteurized milk and milk products are consumed [34,40].

*Enterobacteriaceae* are also associated with mastitis [41]. We isolated a relatively high proportion of members of the Enterobacteriaceae family (55.5%) in our study, which is comparable to a previous report of the isolation of 64.5% *Enterobacteriaceae* from milk samples of goats with mastitis in Thika East subcounty [21]. Coliforms, represented by *Klebsiella* spp. (20.1%), *Escherichia* spp. (4.0%), *Citrobacter* spp. (4.0%), and *Enterobacter* spp. (3.3%), have been identified as the main cause of environmental mastitis in small ruminants. Coliforms are normally associated with environmental contamination of the udder and the establishment of intramammary infection [31]. The presence of coliform bacteria could be linked to poor hygiene practices in dairy goat farms. Poor hygiene is also reflected by an increase in the prevalence of subclinical mastitis (72.7%) recorded in this study, compared with 50.9% previously reported in Thika East subcounty. In addition, the frequency of isolation of different bacteria from goat milk is strongly associated with the overall hygiene status as well as the management strategy of the farms [42].

Although not listed as a major etiological agent, *Acinetobacter* spp. have also been reported in cases of subclinical mastitis in goats. We isolated a higher proportion (16.1%) of *Acinetobacter* spp. in our study compared with that reported in goats in Nigeria (3%) [34]. *Yersinia* spp. have not been associated with subclinical mastitis in goats; however, they have been widely isolated from raw goat milk [43,44].

The high resistance to penicillin G, cephalosporins, and cephamycin observed in our study was unforeseen. However, our findings corroborate with several other studies of dairy goats that reported high resistance to penicillin and cephalosporins of the bacterial isolates causing mastitis [13,36,37]. In addition, we observed cross-resistance among the bacterial isolates causing subclinical mastitis in goats. Similar results showing cross-resistance to penicillin and other β-lactams have been observed in goats [45] and cows [46]. The bacterial resistance mechanism to β-lactam antibiotics involves the production of β-lactamases and the synthesis of modified penicillin-binding proteins that inactivate β-lactam antibiotics by hydrolyzing the amide bond of the four-membered β-lactam ring, preventing drug binding [12,16]. The primary mechanism of bacterial resistance to β-lactam antibiotics is the production of β-lactamases, such as penicillinases, cephalosporinases, broad-spectrum β-lactamases, ESBLs, and carbapenemases [16]. These enzymes hydrolyze penicillins (including benzylpenicillin, also known as penicillin G), third-generation cephalosporins (including cefotaxime and ceftazidime), aztreonam, cephamycins (including cefoxitin), and carbapenems, but are inhibited by β-lactamase inhibitors, such as clavulanic acid [16]. At present, antibiotic therapy is the recommended treatment option for clinical and subclinical mastitis, and β-lactams are among the antibiotics used for mastitis treatment in Kenya. However, antibiotic resistance has spread over the years, mostly due to farmers initiating antibiotic therapy for mastitis treatment before conducting an antibiotic susceptibility test [47]. The high resistance to β-lactam antibiotics is concerning because limited therapeutic options are available for the treatment of such infections compounded with difficulty in controlling these bacteria due to their ability to rapidly spread and transfer the resistance phenotype.

Hygiene is a major contributing factor to the spread of resistance in farms [48,49]. In our study, hygiene was defined by pre- and post-milking practices and the cleaning schedule of the goats’ housing structure. We isolated a higher proportion of antibiotic-resistant bacteria isolated from goats whose farmers used water only and reusable towels during pre- and post-milking compared with those who used water, disinfectant, and reusable towels. This observation is probably due to the bactericidal effect of disinfectant [50]. The use of disinfectant during milking has been recommended as a control strategy to eradicate and prevent the spread of antibiotic-resistant bacteria in dairy farms [50]. However, the occurrence of antibiotic-resistant bacteria among goats whose farmers used disinfectant and reusable towels during milking was probably due to the use of reusable towels that might have been contaminated or not cleaned properly or due to other unknown factors. Furthermore, we found that hygiene practices during milking were significantly associated with the occurrence of resistance. However, hygiene in the goat housing structures was not significantly associated with the occurrence of resistance, which was probably due to the small sample size used in our study or other unknown factors. In addition, mastitis history, previous antibiotic treatment, parity, and age did not significantly impact the occurrence of resistance, and the observed variability can be attributed to different management practices in the farms or other unknown factors.

We did not phenotypically detect any ESBL producers. However, a negative synergy test for the phenotypic detection of ESBLs does not necessarily indicate that the bacteria are negative for ESBL production. False-negative ESBL results can be attributed to the overproduction of AmpC β-lactamases, inhibitor-resistant TEM β-lactamases, or TEM and SHV β-lactamases in ESBL-producing bacteria [30,51]. Therefore, there is a need for genotypic detection of ESBLs in bacterial isolates that show resistance to β-lactam antibiotics.

## Conclusion

We found a high prevalence of subclinical mastitis in the sampled dairy goats in Thika subcounty. The etiological agents causing subclinical mastitis are of zoonotic importance and are resistant to β-lactam antibiotics. This poses a health risk to humans, especially when raw milk and raw milk products are consumed. Hygiene during milking contributes to the occurrence of resistance to β-lactam antibiotics. More stringent measures, such as good animal husbandry and milking practices, must be adopted to control mastitis and prevent the spread of resistance to β-lactam antibiotics. Further studies should be undertaken to determine transmission dynamics between humans and livestock and identify novel intervention strategies.

## Data Availability

The raw data used to support the findings of this study are available from the corresponding author on request.

## Authors’ Contributions

IMO, JK, and NM involved in the conception of the research idea. IMO and JK planned the study design. IMO performed data collection, sample collection, and laboratory work. IMO and DK contributed in statistical analysis and interpretation of the data results. JK and DK provided laboratory guidance. IMO drafted the manuscript. JK, DK, and NM corrected the manuscript. All the authors read and approved the final manuscript.
